# Identification of Hub Genes in Liver Hepatocellular Carcinoma Based on Weighted Gene Co-expression Network Analysis

**DOI:** 10.1007/s10528-024-10803-8

**Published:** 2024-04-29

**Authors:** Jiawei Sun, Zizhen Zhang, Jiaru Cai, Xiaoping Li, Xiaoling Xu

**Affiliations:** 1 Shulan International Medical College, Zhejiang Shuren University, Hangzhou, 31005 China; 2 Key Laboratory of Carcinogenesis and Translational Research (Ministry of Education/Beijing), Department of Gastrointestinal Oncology, Peking University Cancer Hospital & Institute, Beijing, 100142 China

**Keywords:** Weighted gene co-expression network analysis, Bioinformatics analysis, Liver hepatocellular carcinoma, Hub gene, Protein–protein interaction

## Abstract

Liver hepatocellular carcinoma (LIHC) is a malignant cancer with high incidence and poor prognosis. To investigate the correlation between hub genes and progression of LIHC and to provided potential prognostic markers and therapy targets for LIHC. Our study mainly used The Cancer Genome Atlas (TCGA) LIHC database and the gene expression profiles of GSE54236 from the Gene Expression Omnibus (GEO) to explore the differential co-expression genes between LIHC and normal tissues. The differential co-expression genes were extracted by Weighted Gene Co-expression Network Analysis (WGCNA) and differential gene expression analysis methods. The Genetic Ontology (GO) and Kyoto Encyclopedia of Genes and Genomes (KEGG) were carried out to annotate the function of differential genes. Then the hub genes were validated using protein-protein interaction (PPI) network. And the expression level and prognostic analysis were performed. The probable associations between the expression of hub genes and both tumor purity and infiltration of immune cells were explored by TIMER. A total of 68 differential co-expression genes were extracted. These genes were mainly enriched in complement activation (biological process), collagen trimer (cellular component), carbohydrate binding and receptor ligand activity (molecular function) and cytokine − cytokine receptor interaction. Then we demonstrated that the 10 hub genes (CFP, CLEC1B, CLEC4G, CLEC4M, FCN2, FCN3, PAMR1 and TIMD4) were weakly expressed in LIHC tissues, the qRT-PCR results of clinical samples showed that six genes were significantly downregulated in LIHC patients compared with adjacent tissues. Worse overall survival (OS) and disease-free survival (DFS) in LIHC patients were associated with the lower expression of CFP, CLEC1B, FCN3 and TIMD4. Ten hub genes had positive association with tumor purity. CFP, CLEC1B, FCN3 and TIMD4 could serve as novel potential molecular targets for prognosis prediction in LIHC.

**Supplementary Information:**

The online version contains supplementary material available at 10.1007/s10528-024-10803-8.

## Introduction

Liver hepatocellular carcinoma (LIHC) is one of the most common malignancies worldwide, ranking sixth for incidence and third for mortality (Kulik and El-Serag 2019; Sung et al. 2021). Based on the global cancer statistics report, primary liver cancer ranked as the sixth most frequently diagnosed cancer and the third leading cause of cancer-related mortality globally in 2020, LIHC represents 75-85% of all liver cancer cases. (Sung et al. 2021). Chronic infection with hepatitis B virus (HBV) or hepatitis C virus (HCV), aflatoxin-contaminated foods, heavy alcohol intake, excess body weight, type 2 diabetes, and smoking are main risk factors for LIHC (Kulik and El-Serag 2019; Yang et al. 2019). The commonly used therapies for LIHC include hepatectomy, liver transplantation and ablative therapy(Chakraborty and Sarkar 2022; Chen et al. 2020). Although there are many new methods for the diagnosis and treatment of LIHC, the prognosis is still poor due to high rates of recurrence and metastasis. An important reason for poor prognosis is the lack of an accurate prognosis grading system and an effective early screening method for LIHC. Therefore, it is reasonable to evaluate the value of potential biomarkers in the diagnosis and treatment of LIHC.

With the development of genome technology, a variety of tools(Langfelder and Horvath 2008; Newman et al. 2015; van Ijzendoorn et al. 2019) have been used for the study of the molecular mechanism of diseases and biomarker identification. Weighted gene co-expression network analysis (WGCNA) is a novel bioinformatics approach used to describe patterns of gene association between different samples. It can cluster genes and form modules by similar gene expression patterns and analyze the relationship between modules and specific features to identify candidate biomarker genes (Pei et al. 2017). WGCNA is helpful in understanding the molecular mechanisms of cancer and identifying reliable biomarkers for more effective diagnosis, prognosis and treatment.

In this investigation, we comprehensively screened pivotal central genes implicated in LIHC, thereby establishing a robust foundation for the analysis of differentially co-expressed genes. This framework facilitates a deeper understanding of the etiological factors and potential molecular mechanisms underlying LIHC pathogenesis.

## Methods

### Data Processing from TCGA and GEO Database

In order to screen the differential expression genes between LIHC and normal control, we obtained the data of gene expression profiles in TCGA (https://portal.gdc.cancer.gov/projects/TCGA-LIHC) (including 50 normal tissues and 374 tumor tissues) and GEO (https://www.ncbi.nlm.nih.gov/gds) GSE54236 (including 77 adjacent nontumorous samples and 78 LIHC samples; platform: GPL6480), After removing repetitive probes of the same gene, an interpreted gene expression profile of 18,076 genes was obtained for the following analysis.

### Co-expression Network Construction by WCGNA

The *WGCNA* package in R was employed to construct a gene co-expression network using the gene expression data profiles of TCGA-LIHC and GSE54236 (Chen et al. 2017; Langfelder and Horvath 2008). The package includes functions for network construction, module detection, gene selection, calculations of topological properties, data simulation, visualization, and interfacing with external software(Langfelder and Horvath 2008). First, the expression levels of individual transcripts were transformed to generate a similarity matrix based on Pearson correlation values between paired genes. Then, the similarity matrix is transformed into the adjacency matrix. β = 3 and 20 were selected as the soft threshold power. The adjacency matrix is made from the following formula: ${a}_{ij}=| {c}_{ij} {|}^{\beta }$ Next the adjacency matrix transformed to corresponding topological overlap matrix (TOM). The genes were divided into different modules by dynamic mixed cutting method, and the minimum module size cut-off value of 30 was simulated.

### Identification of DEGs and Validation of Co-expression Modules

To explore differentially expressed genes (DEGs) in TCGA-LIHC and GSE54236 data sets, the R-package *limma*, an integrated solution of analysis for RNA-Sequencing and microarray data, was used to filter out DEGs in LIHC(Li et al. 2020). |logFC| ≥1.0 and *P* < 0.05 were defined for screening DEGs. A volcano plot using the R package *ggplot2* was constructed to reveal the differentially expressed genes. The overlapped genes between the DEGs extracted from the co-expression network were presented by Venn diagram.

### Function Enrichment Analysis

In order to conduct the Genetic Ontology (GO) enrichment and Kyoto Encyclopedia of Genes and Genomes (KEGG) pathway analysis, R Package *clusterProfiler* was used to explore the functions between the selected genes(“The Gene Ontology Resource: 20 years and still GOing strong,” 2019). Statistical significance of GO terms or KEGG pathways was considered as *P* < 0.05. The GO annotation contains biological process (BP), cellular component (CC) and molecular function (MF).

### Construction of PPI Network and Screening of Hub Genes

To construct a PPI network of the identified differentially expressed genes (DEGs), the online database Search Tool for the Retrieval of Interacting Genes (STRING)(Szklarczyk et al. 2019) was used. Genes with confidence score ≥ 0.4 were chosen to build a network model by Cytoscape visualization software (v3.7.2)(Doncheva et al. 2019; Shannon et al. 2003). Maximal Clique Centrality (MCC), an effective way to find a hub node, was calculated by CytoHubba (Chin et al. 2014), and the 10 genes with the highest MCC values were identified as the hub genes.

### Human LIHC Samples

This study included 10 pairs of LIHC tissue and paired adjacent tissue samples, which were obtained from patients who underwent liver surgery at Shulan Hospital of Zhejiang Shuren University. Ethical approval for human subjects was obtained from the research ethics committee of Zhejiang Shuren University (approval number: 2024/041), and informed consent was obtained from each patient.

### RNA Extraction and qRT-PCR

Total RNA from each sample was extracted applying TRIzol reagent (Thermo Fisher, USA). For qRT-PCR, a LightCycler 480 PCR System (Roche, USA) with FastStart Universal SYBR Green Master (Roche, Switzerland) was used. The PCR conditions were: 95 °C pre-denaturation for 30 s, followed by 39 cycles. Each cycle contained 95 °C denaturation for 5 s, 55 °C annealing for 30 s, and 72 °C extension for 30 s. Data were analyzed with the 2 − ΔΔCt method with GAPDH as an internal reference. Supplementary Table S1 listed the primer sequences.

### Validation and Survival Analysis of Hub Genes

First, we used GEPIA2 (http://gepia2.cancer-pku.cn/) (Tang et al. 2019) to verify the expression pattern of hub genes in LIHC tissues and normal tissues from TCGA database. Gene expression data of liver cancer patients with clinical information was downloaded from the International Cancer Genome Consortium (ICGC) (http://icgc.org/), and analyzed to validate expression level of hub genes. Based on data from TCGA database, LIHC patients were divided into two groups according to the median expression level of hub gene. Then Kaplan-Meier survival analysis was performed using survival package in R software to detect the relationship between overall survival (OS) and hub genes. In addition, the relationship between disease-free survival (DFS) and hub gene expression in LIHC patients was determined with the online tool GEPIA2 (Tang et al. 2019). *P* < 0.05 was regarded as the judgment criterion of statistically significant survival-related hub genes.

### Immune Infiltration Analysis TIMER

To find the correlation between the expression of the hub genes and tumor infiltrating immune cell subtypes including B cells, CD4^+^ T cells, CD8^+^ T cells, neutrophils, macrophages, and dendritic cells, we utilized the online comprehensive tool Tumor Immune Estimation Resource (TIMER, http://timer.cistrome.org/), an online systematic analysis database containing 10,897 samples across 32 cancer types from the TCGA database (Li et al. 2016, 2017, 2020a, b).

## Results

### Weighted Gene Co-expression Modules Construction

**Fig. 1:**
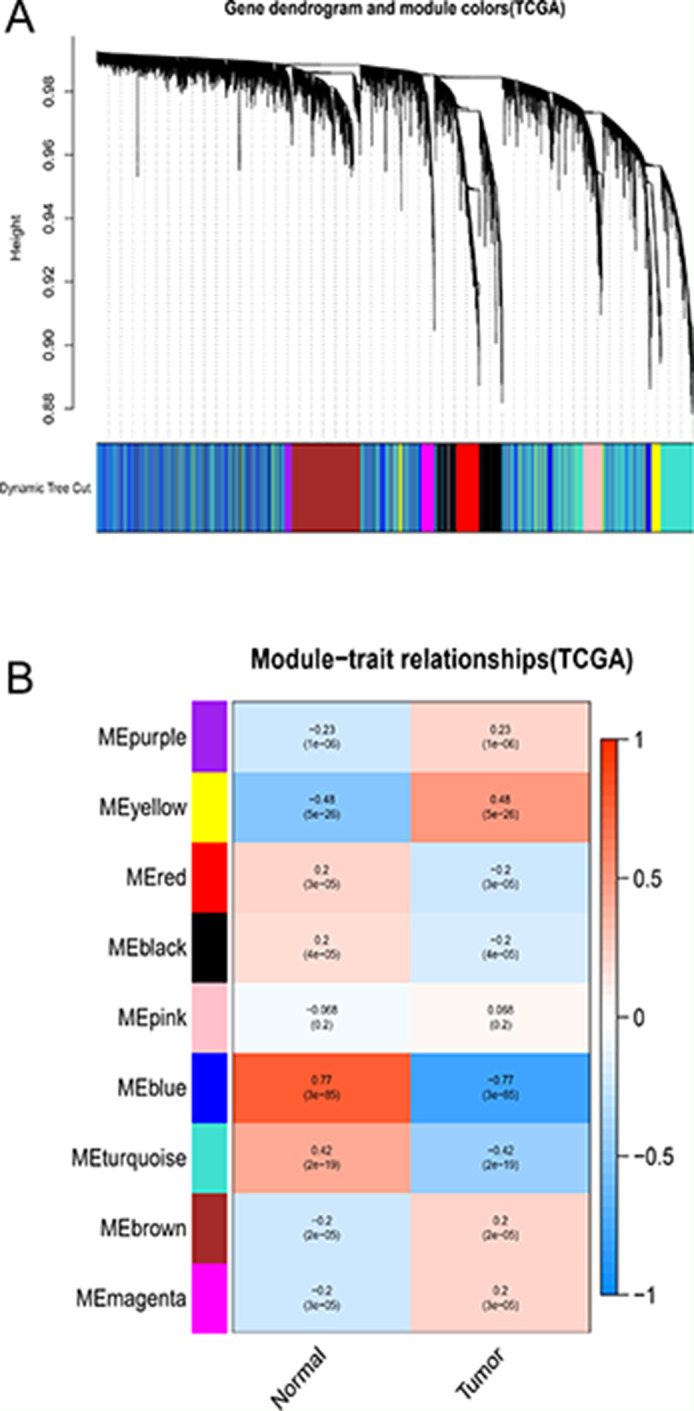
Identification of key modules correlated with clinical information in the TCGA-LIHC dataset through WGCNA. (**A**) Cluster dendrogram of all co-expression network modules based on a dissimilarity measure (1-TOM). Each co-expression module was represented by a color. (**B**) Heatmap of module-trait relationships. Each cell contains the corresponding correlation and *P*-value

**Fig. 2:**
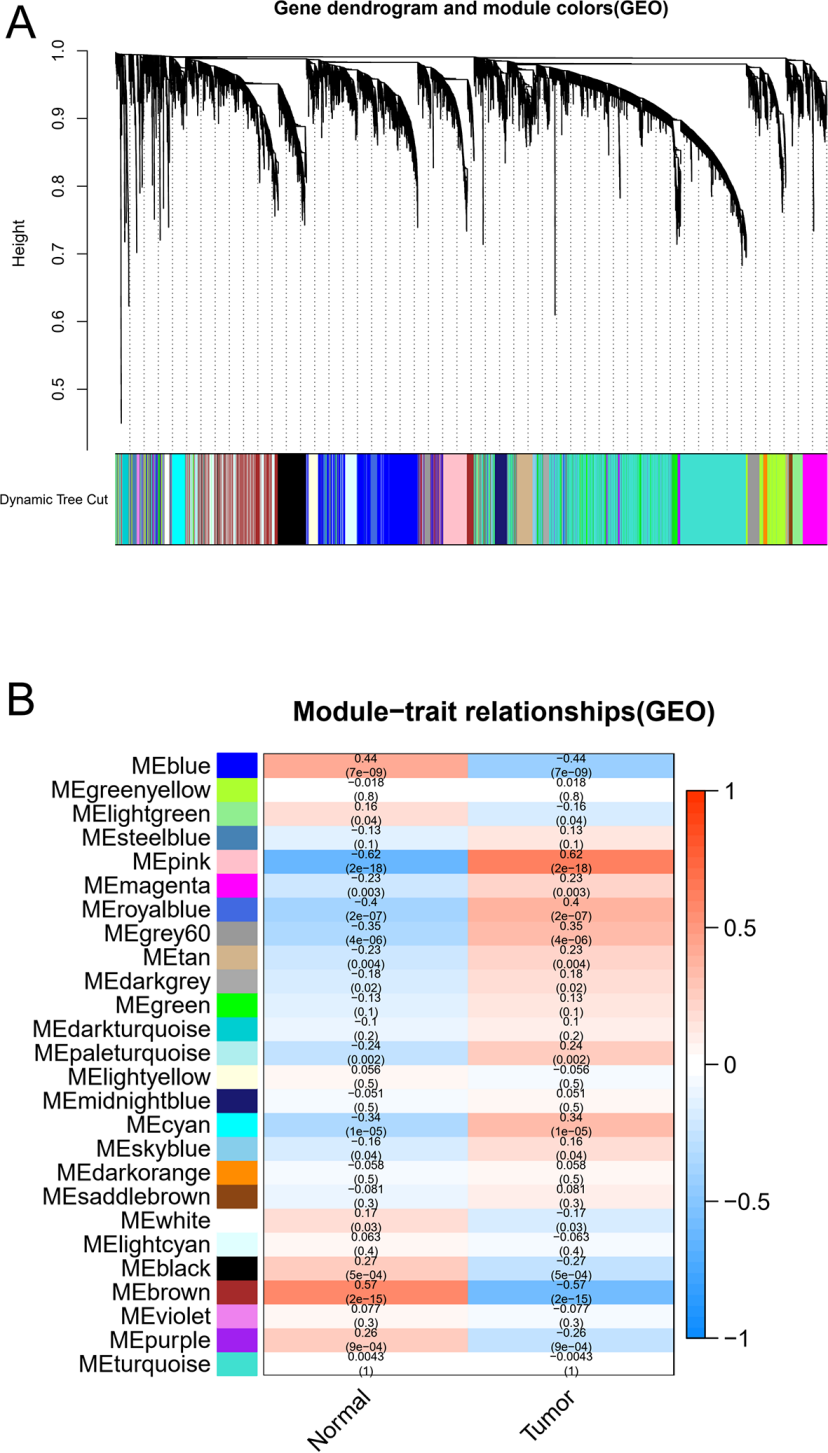
Identification of key modules correlated with clinical information in the GSE54236 dataset through WGCNA. (**A**) Cluster dendrogram of all co-expression network modules based on a dissimilarity measure (1-TOM). Each co-expression module was represented by a color. (**B**) Heatmap of module-trait relationships. Each cell contains the corresponding correlation and P-value

Using the WGCNA package, we constructed the gene expression data profiles of TCGA-LIHC and GSE54236 to gene co-expression networks. In the present study, each module was represented by a color, we emerged 9 modules in the TCGA-LIHC (Fig. 1A) and 27 modules in the GSE54236 (Fig. 2A) from the analysis. The results obtained from the preliminary analysis of the module-trait relationships showed that the blue module in the TCGA-LIHC (Fig. 1B) and brown module in the GSE54236 (Fig. 2B) were found to have the strongest association with normal tissues (blue module: *r* = 0.77, *p* = 3e-85; brown module: *r* = 0.57, *p* = 2e-15).

### Identification of Genes between the DEGs and Co-expression Modules

**Fig. 3:**
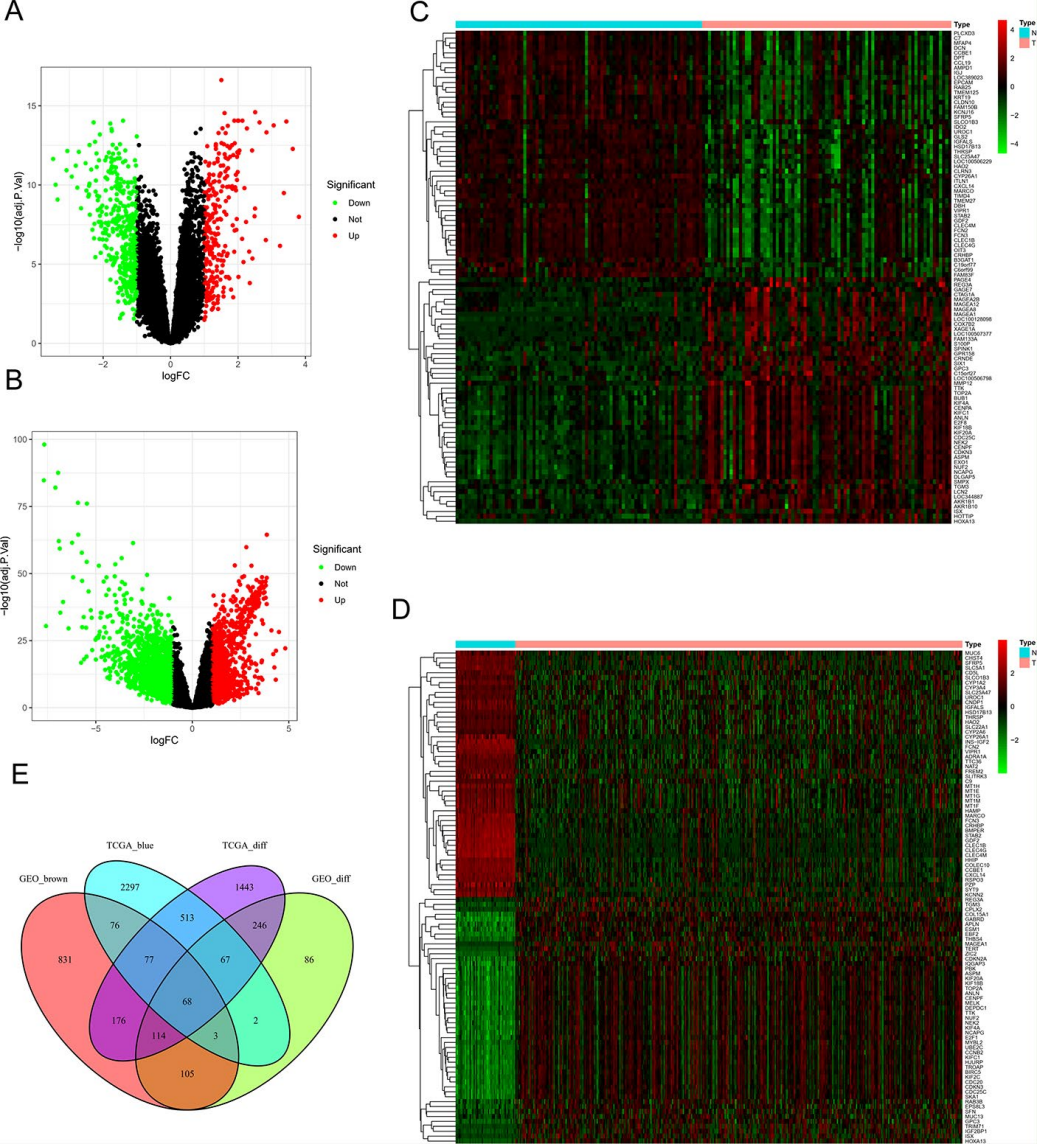
Identification of differentially expressed genes (DEGs) among the TCGA and GSE54236 datasets of LIHC. (**A**) Volcano plot of DEGs in the TCGA dataset. (**B**) Volcano plot of DEGs in the GSE54236 dataset. |logFC| ≥ 1.0 and adj.*P* < 0.05 were defined as the cut-off criteria Green and red indicated low and high expression in LIHC, respectively. Black indicated that those genes no difference between LIHC and normal tissues. (**C**) Heatmap of DEGs in the TCGA dataset. (**D**) Heatmap of DEGs in the GSE54236 dataset. (**E**) The Venn diagram of 68 overlapping genes among two DEG lists and two co-expression modules

We identified 2704 DEGs in TCGA-LIHC **(**Fig. 3A) and 691 DEGs in GSE54236 (Fig. 3B) using volcano plot. And 3103 and 1450 co-expression genes were separately selected in the blue module of TCGA-LIHC and brown module of GSE54236. A heatmap is a simple yet effective way to compare the content of multiple major gene lists. The heatmap showed the distribution of the top 100 DEGs in TCGA-LIHC and GSE54236 (Fig. 3C, D). To overlap the gene in two co-expression modules and DEG list, a total of 68 overlapping genes were performed by Venn diagram (Fig. 3E).

### Functional Enrichment Analyses

**Fig. 4:**
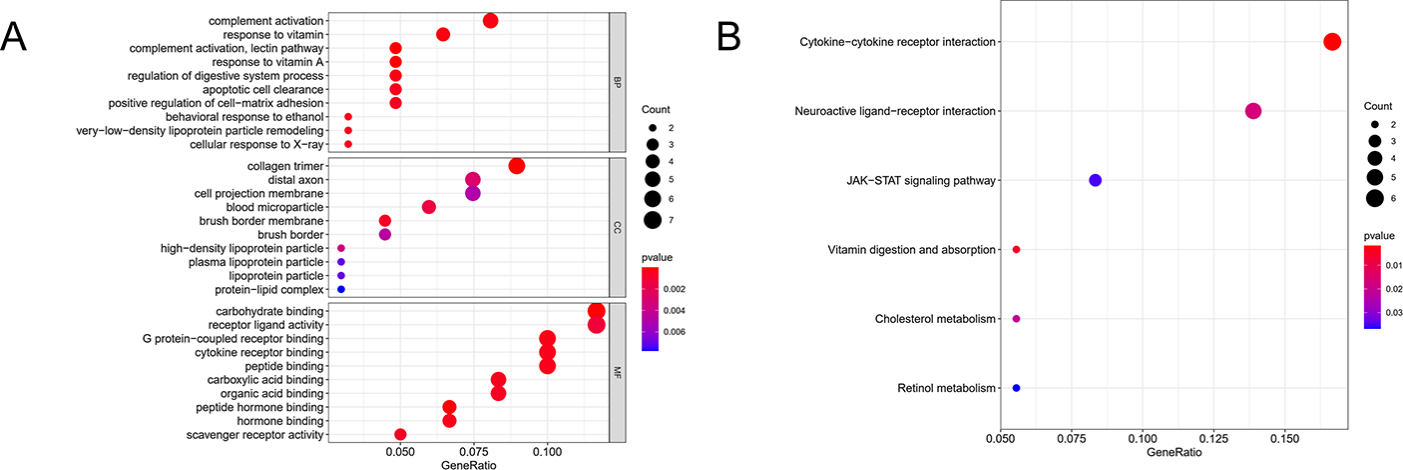
GO functional and KEGG pathway enrichment analysis for the candidate genes. (**A**) Biological process (BP), cellular component (CC), and molecular function (MF) of GO enrichment. (**B**) KEGG pathway enrichment analysis. The color indicates the adjusted P-values, and the size of the spots indicates the gene number

To reveal the potential functions of the 68 genes, we divided genes into several enriched gene sets based on the Gene Ontology (GO) analysis. In the biological process (BP) analysis, the 68 genes mainly involved in complement activation. In the result of the cellular component (CC), these genes were mainly enriched in collagen trimer. The molecular function (MF) of 68 genes was mainly enriched in carbohydrate binding and receptor ligand activity (Fig. 4A). Meanwhile we conducted Kyoto Encyclopedia of Genes and Genomes (KEGG) analysis, showing that cytokine − cytokine receptor interaction was widely related to the 68 genes (Fig. 4B).

### Construction of the PPI Network

**Fig. 5:**
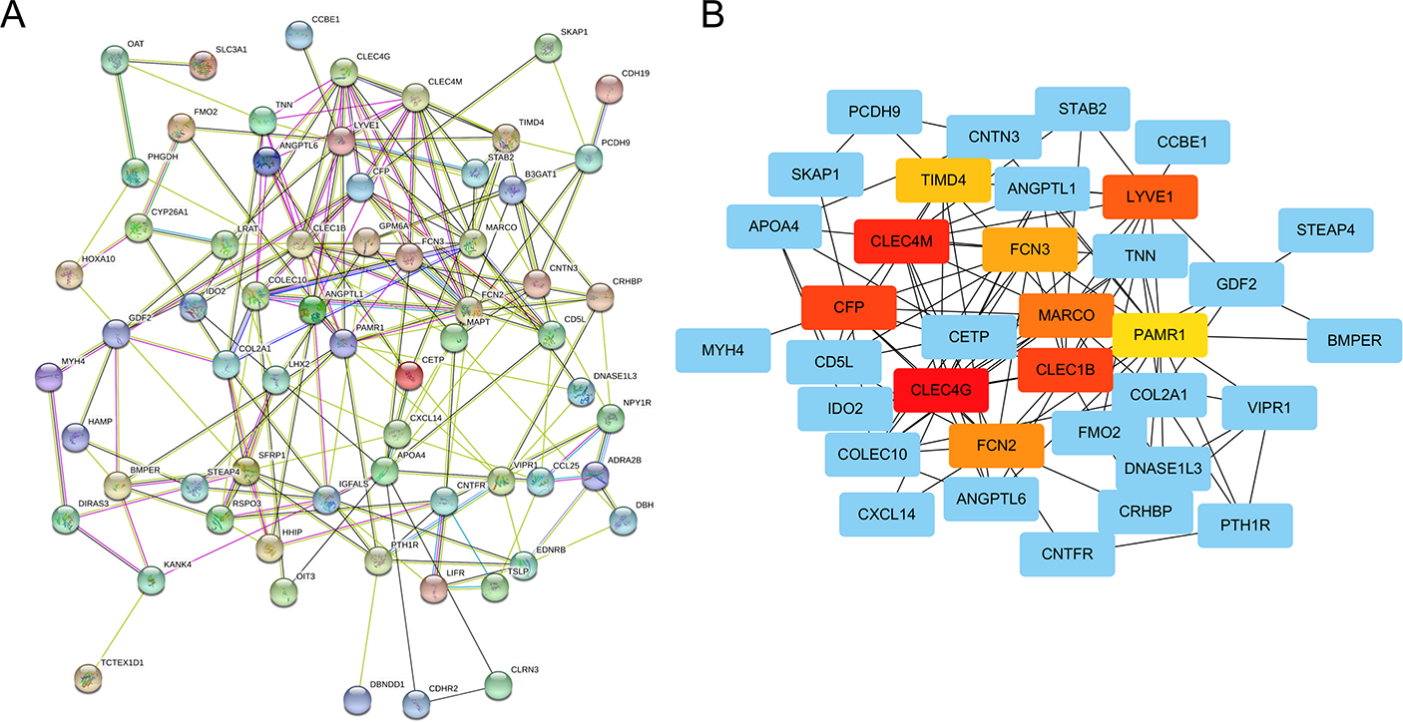
The enrichment analysis of the candidate genes by protein interaction (PPI) network. (**A**) PPI network. (**B**) Identification of 10 hub genes from PPI network utilizing maximal clique centrality (MCC) algorithm. Red nodules represent MCC high genes and yellow nodules represent MCC low genes

In order to find the hub genes, we performed the PPI analysis of overlapped genes the utilized the “String” website tool (Fig. 5A). Among the 11 topological analysis methods provided by CytoHubba, MCC performed better in predicting the accuracy of essential proteins from PPI networks. Using the MCC algorithm of CytoHubba plugin, the top 10 highest-scored genes, including CFP (Complement Factor Properdin), CLEC1B (C-type lectin domain family 1 member B), CLEC4G (C-type lectin domain family 4 member G), CLEC4M (C-type lectin domain family 4 member M), FCN2(Ficolin 2), FCN3(Ficolin 3), LYVE1(Lymphatic Vessel Endothelial Hyaluronan Receptor 1), MARCO(Macrophage Receptor With Collagenous Structure), PAMR1(Peptidase Domain Containing Associated With Muscle Regeneration 1), TIMD4(T cell immunoglobulin and mucin domain containing 4) were selected as the hub genes from the PPI network (Fig. 5B).

### Validation and Survival Analysis of Hub Genes and Expression of Hub Genes

**Fig. 6:**
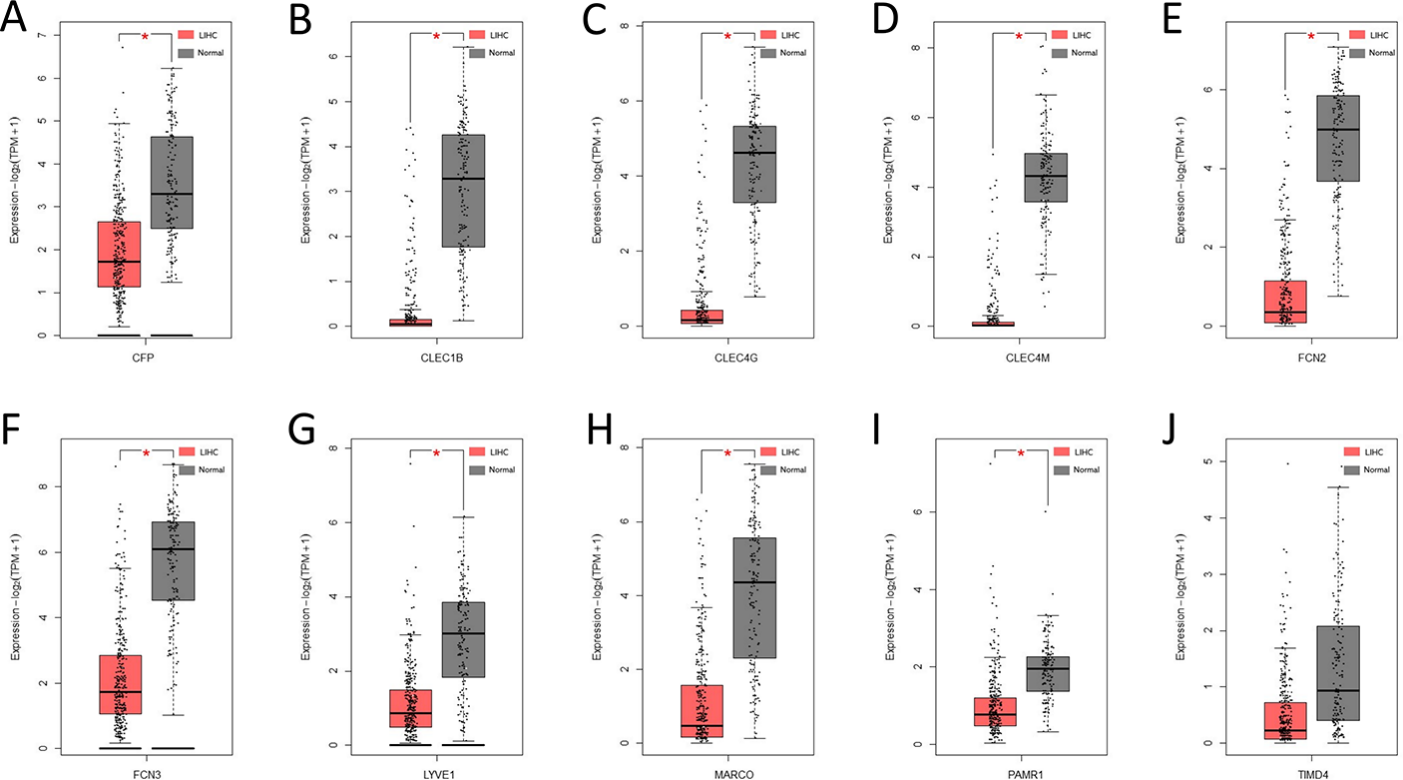
Expression levels of the 10 hub genes among LIHC and normal tissues from the TCGA dataset. (**A**) CFP, (**B**) CLEC1B, (**C**) CLEC4G, (**D**) CLEC4M, (**E**) FCN2, (**F**) FCN3, (**G**) LYVE1, (**H**) MARCO, (**I**) PAMR1, (**J**) TIMD4. *P < 0.05. *P* < 0.05 denotes significance

**Fig. 7:**
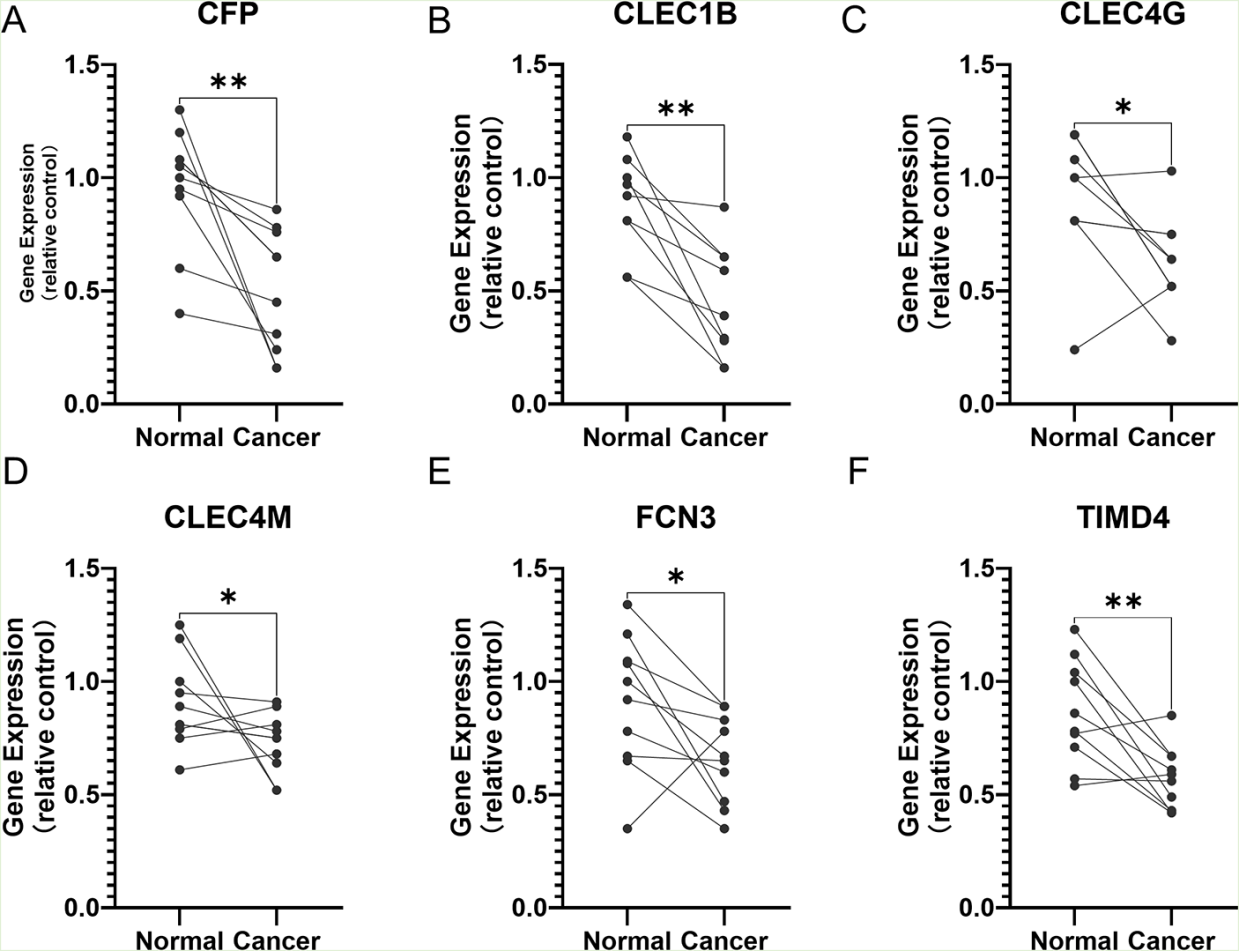
The expression of hub genes in LIHC patients. (**A**) CFP, (**B**) CLEC1B, (**C**) CLEC4G, (**D**) CLEC4M, (**E**) FCN3, (**F**) TIMD4. (*P < 0.05, **P < 0.01)

**Fig. 8:**
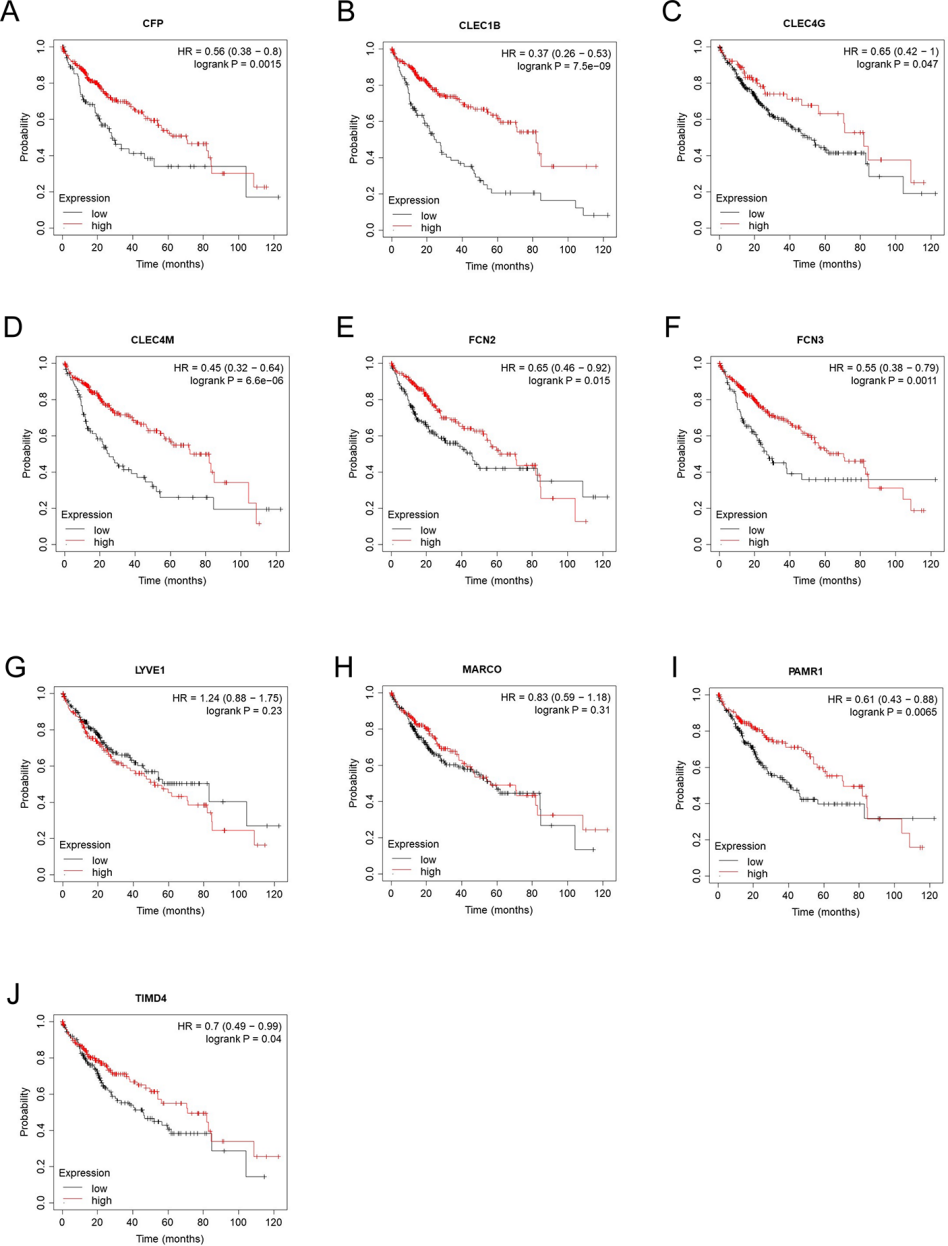
Overall survival (OS) analysis of hub genes in LIHC patients. (**A**) CFP, (**B**) CLEC1B, (**C**) CLEC4G, (**D**) CLEC4M, (**E**) FCN2, (**F**) FCN3, (**G**) LYVE1, (**H**) MARCO, (**I**) PAMR1, (**J**) TIMD4. *P* < 0.05 denotes significance

**Fig. 9:**
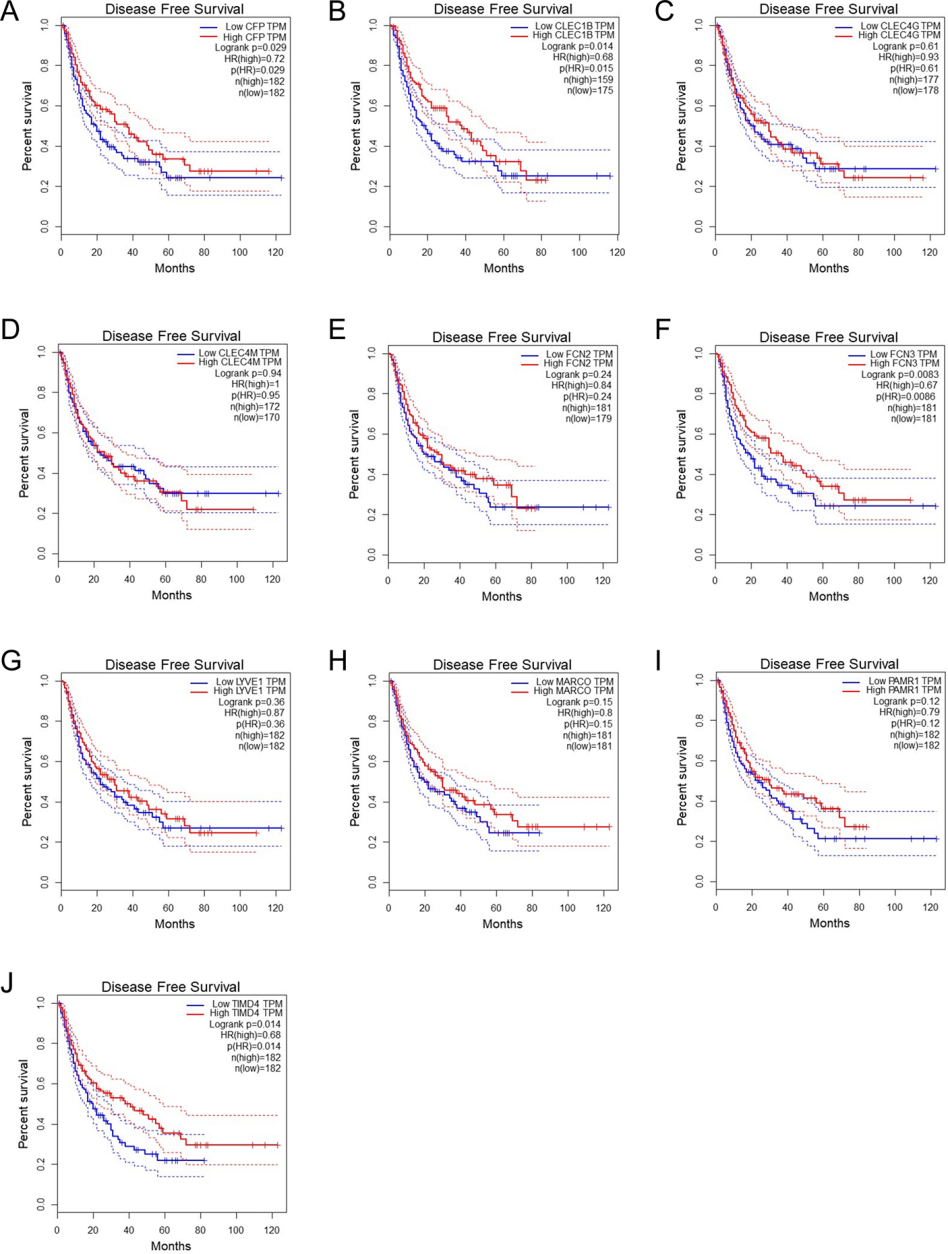
Disease-free survival (DFS) analysis of hub genes in LIHC patients. (**A**) CFP, (**B**) CLEC1B, (**C**) CLEC4G, (**D**) CLEC4M, (**E**) FCN2, (**F**) FCN3, (**G**) LYVE1, (**H**) MARCO, (**I**) PAMR1, (**J**) TIMD4. *P* < 0.05 denotes significance

Then we verified the expression levels of the hub genes among patients from the TCGA and ICGC database. All 10 hub genes were found to be significantly downregulated in LIHC compared to normal tissue (Fig. 6; Table 1). More convincingly, the qRT-PCR results of clinical samples showed that compared with adjacent tissues six genes (CFP, CLEC1B, CLEC4G, CLEC4M, FCN3, TIMD4) were significantly downregulated in LIHC patients (*P* < 0.005) (Fig. 7). However, the other for genes (LYVE1, MARCO, PAMR1, FCN2) did not show significant trends. The OS and DFS analyses were performed by Kaplan Meier plot to identify the effect of hub genes on the prognosis from PPI network complexes and WGCNA modules. The results showed that low expression of CFP (Fig. 8A), CLEC1B (Fig. 8B), CLEC4G (Fig. 8C), CLEC4M (Fig. 8D), FCN2 (Fig. 8E), FCN3 (Fig. 8F), PAMR1 (Fig. 8I) and TIMD4 (Fig. 8J) was associated with poor OS in LIHC patients (*P* < 0.05). Besides, among the hub genes, the lower expression level of CFP (Fig. 9A), CLEC1B (Fig. 9B), FCN3 (Fig. 9F) and TIMD4 (Fig. 9J) was significantly associated with worse DFS of LIHC patients (*P* < 0.05).

**Table 1:** Expression of the Hub gene in LIHC and adjacent tissues from the ICGC database

Gene	Type	Expression log2(TPM + 1)	*P* value
CFP	LIHC	1.49 ± 0.89	8.95E-98
	Adjacent	3.89 ± 0.76	
CLEC1B	LIHC	0.59 ± 0.96	4.41E-107
	Adjacent	3.88 ± 1.05	
CLEC4G	LIHC	0.75 ± 1.18	1.20E-125
	Adjacent	4.71 ± 0.98	
CLEC4M	LIHC	0.52 ± 1.14	8.52E-110
	Adjacent	4.16 ± 1.10	
FCN2	LIHC	0.96 ± 1.30	4.77E-115
	Adjacent	4.90 ± 1.05	
FCN3	LIHC	2.091 ± 1.69	1.03E-101
	Adjacent	6.426 ± 1.13	
LYVE1	LIHC	1.26 ± 0.93	1.95E-62
	Adjacent	3.42 ± 1.10	
MARCO	LIHC	1.19 ± 1.42	7.06E-80
	Adjacent	4.88 ± 1.51	
PAMR1	LIHC	0.83 ± 0.63	2.70E-61
	Adjacent	2.10 ± 0.62	
TIMD4	LIHC	0.74 ± 0.84	9.22E-40
	Adjacent	2.23 ± 1.06	

### Association between Hub Genes with Immune Infiltration Level

**Fig. 10:**
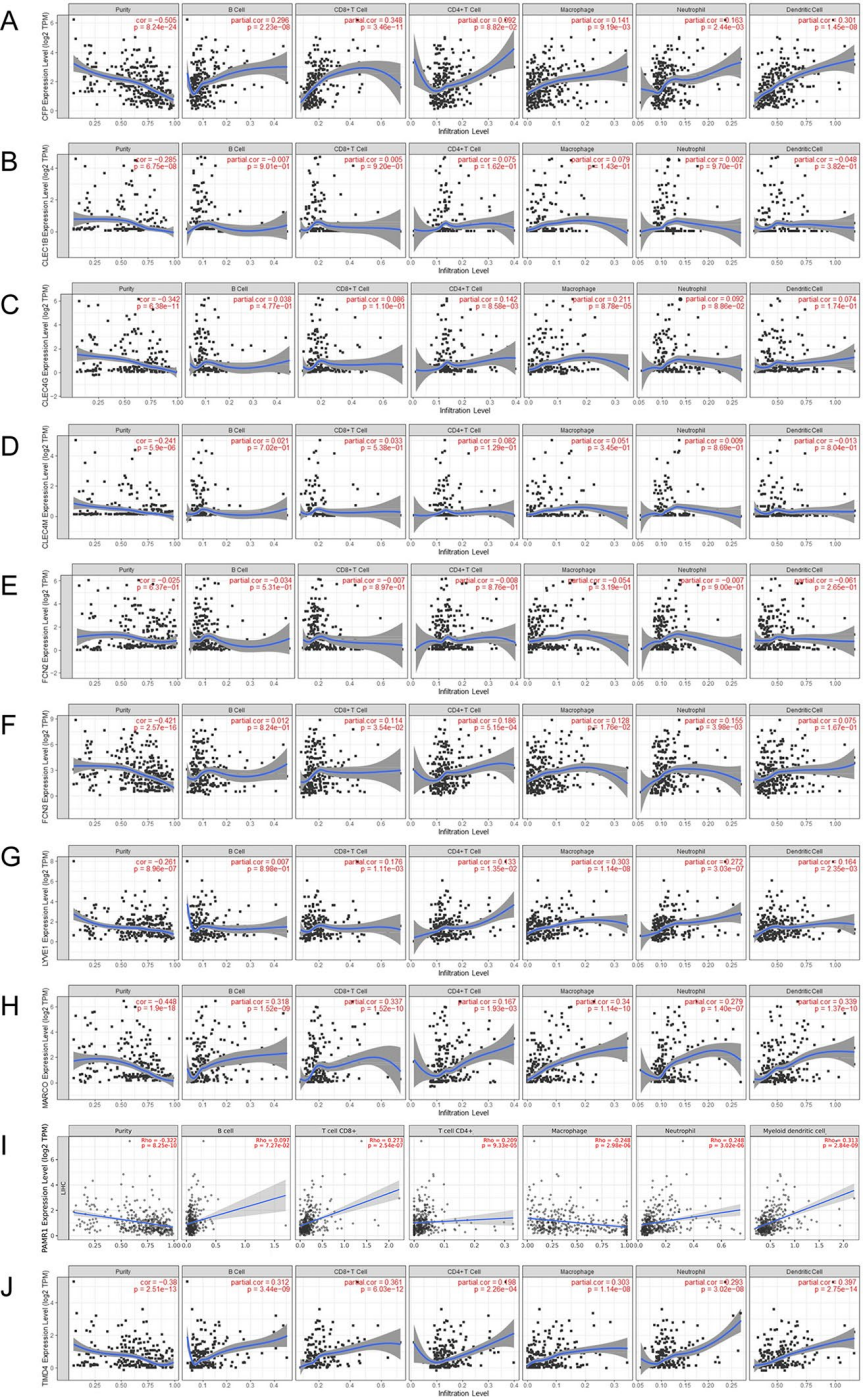
Association between expression of hub genes and immune infiltration in LIHC. (**A**) CFP, (**B**) CLEC1B, (**C**) CLEC4G, (**D**) CLEC4M, (**E**) FCN2, (**F**) FCN3, (**G**) LYVE1, (**H**) MARCO, (**I**) PAMR1, (**J**) TIMD4. Each dot represents a sample from the TCGA-LIHC dataset. *P* < 0.05 denotes significance

Infiltrating immune cells are an important part of tumor microenvironment besides tumor cells and stromal cells. The online search tool TIMER was utilized to explore probable associations between the expression of hub genes and both tumor purity and infiltration of immune cells. All those 10 hub genes had positive association with tumor purity (Fig. 10), while no or weak correlation with B cell infiltration, CD4 + T cells, CD8 + T cells, neutrophils, macrophages, and dendritic cells (Fig. 10).

## Discussion

LIHC remains one of the most malignant cancers with poor prognosis. WGCNA has been used to explore biomarkers related to pathogenesis, diagnosis and prognosis of LIHC. Immune cell infiltration related biomarkers in LIHC including CCL5, CXCR6, CD3E and LCK were identified using WGCNA(Bai et al. 2020; Gao et al. 2023). Another study found that 13 hub genes (GTSE1, PLK1, NCAPH, SKA3, LMNB2, SPC25, HJURP, DEPDC1B, CDCA4, UBE2C, LMNB1, PRR11, and SNRPD2) had high correlation with histologic grade in LIHC by analyzing TCGA LIHC dataset(Gu et al. 2020). While our study provides a more comprehensive and innovative idea from various sources of data.

Through a comprehensive bioinformatics analysis, a total of 68 significant DEGs were identified in the TCGA-LIHC and GSE54236 datasets. These genes were primarily clustered in functional categories related to complement activation, collagen trimer, receptor-ligand activity, and cytokine-cytokine receptor interaction. The complement system, an ancient defense mechanism predating adaptive immunity, and its activation are known to play a crucial role in tumor-promoting inflammation (Afshar-Kharghan 2017; Senent et al. 2022). Chronic infection with HBV or HCV is a major risk factor for LIHC, with complement activation contributing to the progression from inflammation to tumor development. Pathways involving cytokines and ligand-receptor interactions are also implicated in the pathogenesis of early-stage LIHC(Ma et al. 2022; Molina et al. 2019). Collagen, a key component of the extracellular matrix, with collagen trimer serving as a biomarker for cancer metastasis(Xu et al. 2019). By constructing a PPI network, we identified 10 hub genes, including CFP, CLEC1B, CLEC4G, CLEC4M, FCN2, FCN3, LYVE1, MARCO, PAMR1, and TIMD4. The expression levels of these hub genes were down-regulated in LIHC compared to normal controls. Notably, the decreased expression of CFP, CLEC1B, FCN3, and TIMD4 was significantly associated with poor OS and DFS in LIHC patients. Analysis using the TIMER revealed that these hub genes were predominantly expressed in LIHC cells rather than immune cells, and did not play a role in immune regulation within the tumor microenvironment. These findings suggest that CFP, CLEC1B, FCN3, and TIMD4 may serve as potential therapeutic targets for LIHC and offer valuable insights for prognostic biomarker assessment.

In our study, CFP, CLEC1B, FCN3 and TIMD4 were down-regulated in LIHC tissues compared with normal tissues, and could be strong predictors of poor outcome in LIHC, which may indicate that these genes may function as tumor suppressor genes. Low expression levels of tumor suppressor genes are often linked to a poor prognosis. In addition to their potential involvement in carcinogenesis, there is also a possibility that their low expression contributes to the development of treatment resistance, thereby impacting patient prognosis. However, this is only a conjecture, and we plan to conduct in vitro experiments to further verify this next. The relationship between the four genes and cancer has been confirmed by several previous studies.

CFP (Complement Factor Properdin) is a protein coding gene, and can positively regulate the alternative complement pathway of the innate immune system yielding the elimination of pathogens, apoptotic and necrotic cells (Kemper et al. 2008, 2010). Several studies reported the tumor suppressive effect of CFP on melanoma, breast, stomach and lung cancer(Al-Rayahi et al. 2017; Block et al. 2019; Cui et al. 2021). In stomach and lung cancer, the expression level of CFP was lower than in normal tissues, and low expression level of CFP was associated with poor prognosis (Cui et al. 2021).

CLEC1B (C-type lectin domain family 1 member B) is a novel platelet-associated molecule secreted by activated platelets around tumors(Meng et al. 2021). CLECB1 has been confirmed its inhibitory effect on platelet aggregation and tumor metastasis in colon cancer (Suzuki-Inoue et al. 2007). CLEC1B has been reported to be significantly down-regulated in liver cancer (Critelli et al. 2017). Compared to paired normal tissues, the mRNA and protein levels of CLEC1B were significantly down-regulated in LIHC(Jing et al. 2023).

FCN3 (Ficolin 3) is a secreted lectin that activates the complement pathway (Endo et al. 2011). FCN3 expression was significantly down-regulated in lung cancer tissues compared with matched normal lung tissues, low expression levels of FCN3 have been described as prognostic biomarker for cancer (Jang et al. 2021). LIHC patients were divided into FCN3 high and low expression groups by immunohistochemical staining. patients with high FCN3 expression had a higher overall survival rate than those with low FCN3 expression (*p* = 0.031), which was consistent with our study. And high FCN3 expression in tumor tissue was independently associated with better overall survival (*p* = 0.042)(Chen et al. 2023).

TIMD4 (T cell immunoglobulin and mucin domain containing 4) is a phosphatidylserine receptor that enhances the engulfment of apoptotic cells, and is involved in regulating T-cell proliferation and lymphotoxin signaling (Freeman et al. 2010). As opposed to LIHC, TIMD4 is overexpressed in lung cancer and colorectal cancer, which is correlated with poor prognosis (Dorfman et al. 2010; Tan et al. 2018).

We admit that this study has some limitations and deficiencies. First, this research mainly focuses on data mining and data analysis based on methodology, and the results have not been verified by cytology experiment. Further experiments are needed to better confirm the findings of this study. Our research also has certain limitations for the classification of different subtypes of tumors. Various subtypes of LIHC exhibit distinct clinical presentations, treatment options, and responses to treatment. LIHC can be categorized based on a range of criteria. For instance, common histological morphologies of LIHC include trabecular pattern, pseudoglandular pattern, macrotrabecular, and solid pattern types. The development of LIHC is linked to diverse chronic liver diseases or external exposures. Hepatocellular carcinoma staging can be determined by tumor characteristics or liver function impairment, such as American Joint Committee on Cancer-Tumor Node Metastasis staging (TNM), Cancer of the Liver Italian Program (CLIP) (Kulik and El-Serag 2019; Yang et al. 2019). The correlation between hub genes and LIHC of different etiologies, pathologic features, and clinical stages will be analyzed in greater depth if the analysis is based on a more comprehensive clinical profile of patients. Although our comprehensive bioinformatics analysis identifies potential diagnostic genes for LIHC, it may be biased for patients with different LIHC subtypes.

In conclusion, our employment of WGCNA has provided valuable insights into the progression from normal liver tissue to LIHC. Through this approach, we have identified two modules and ten pivotal hub genes that play critical roles in tumorigenesis. Notably, the decreased expression of CFP, CLEC1B, FCN3, and TIMD4 was significantly associated with poor OS and DFS outcomes in LIHC patients. Furthermore, TIMER for analysis revealed that these hub genes are predominantly expressed in LIHC cells rather than immune cells and do not participate in immune regulation within the tumor microenvironment. These collective findings not only highlight the potential of CFP, CLEC1B, FCN3, and TIMD4 as therapeutic targets for LIHC but also offer valuable insights for improving prognostic biomarker assessment.

## Electronic Supplementary Material

Below is the link to the electronic supplementary material.

Supplementary Material 1

## Data Availability

The datasets generated for this study can be found in the Publicly available datasets were analyzed in this study. This data can be found at: TCGA-LIHC: https://portal.gdc.cancer.gov/projects/TCGA-LIHC GSE54236: https://www.ncbi.nlm.nih.gov/geo/query/acc.cgi?acc=GSE54236 ICGC-LIRI-JP: https://dcc.icgc.org/projects/LIRI-JP.
