# Supplementary material for: Identification of Hub Genes in Liver Hepatocellular Carcinoma Based on Weighted Gene Co-expression Network Analysis
Source: Biochem Genet. 2024 Apr 29;63(3):2120–39. doi: 10.1007/s10528-024-10803-8 (PMC12144059; doi:10.1007/s10528-024-10803-8)
Supplement: Supplementary file 1 — Supplementary Material 1 [file 10528_2024_10803_MOESM1_ESM.docx]

| Target Gene | Primer Sequence (5' -> 3') |
| --- | --- |
| **CFP** |  |
| forward | TGCTCTGCTTCACCCAGTATG |
| reverse | CCACTACGTTTCTGGTAGGCA |
| **CLEC1B** |  |
| forward | AGCGCAATTACCTACAAGGTG |
| reverse | CTTCCCATGTTAAGTTGTGCCT |
| **CLEC4G** |  |
| forward | AGTCCTTTGGGCTGTGATTCT |
| reverse | AGGCGTTTGTCCTCAGCAG |
| **CLEC4M** |  |
| forward | TCCAGAACAACTCCTGCGAG |
| reverse | CACTGGTAGCCCTGAACCT |
| **FCN2** |  |
| forward | AGTGCGGGAGATTCCCTGA |
| reverse | CGGTGTTAAGATCATTGTCCTGG |
| **FCN3** |  |
| forward | GCCCTCCCAGTCTTTTGTGAC |
| reverse | CCTGGAGAGTAAGCTGGTGC |
| **LYVE1** |  |
| forward | AGGCTCTTTGCGTGCAGAA |
| reverse | GGTTCGCCTTTTTGCTCACAA |
| **MARCO** |  |
| forward | CAGCGGGTAGACAACTTCACT |
| reverse | TTGCTCCATCTCGTCCCATAG |
| **PAMR1** |  |
| forward | CCTTGCCAAGAGAGTACACAG |
| reverse | GGCAGACGCACTCAATCTGA |
| **TIMD4** |  |
| forward | ATTGAGTTTTGGTGGCTTTACCT |
| reverse | GAGTAGGGGCACTGGTCTTTC |
| **GAPDH** |  |
| forward | GGAGCGAGATCCCTCCAAAAT |
| reverse | GGCTGTTGTCATACTTCTCATGG |

Supplementary Table 1. Primer sequences used to amplify target genes in human

by real-time RT-PCR.
